# Feasibility of MEG in assessing task-related oscillatory markers and evaluating cognitive changes in recently symptomatic carotid endarterectomy patients: A pilot study

**DOI:** 10.1371/journal.pone.0343689

**Published:** 2026-03-06

**Authors:** Tiia Kukkonen, Sanna Liuha, Juha Leukkunen, Hanna-Maija Lapinkero, Ilmari Rouvinen, Simo Monto, Tiina Parviainen

**Affiliations:** 1 Department of Surgery, Hospital Nova of Central Finland, Wellbeing Services County of Central Finland, Jyväskylä, Finland; 2 Department of Neurology, Hospital Nova of Central Finland, Wellbeing Services County of Central Finland, Jyväskylä, Finland; 3 Center for Interdisciplinary Brain Research, Department of Psychology, Faculty of Education and Psychology, University of Jyväskylä, Jyväskylä, Finland; Athinoula A Martinos Center for Biomedical Imaging, UNITED STATES OF AMERICA

## Abstract

Revascularization ensures oxygen supply to the brain in patients with significant carotid atherosclerosis. The current indication for carotid endarterectomy (CEA) is stroke risk reduction, but it may also impact cognitive functions and their neural basis. Findings on cognitive outcomes after CEA are inconsistent, and the changes in task-related brain electrophysiology are unknown. In this pilot study, we studied the feasibility of using magnetoencephalography (MEG) to follow the associated brain changes during the first postoperative year in five recently symptomatic CEA patients. Head magnetic resonance imaging (MRI), neuropsychological examinations (NPE), and MEG were performed prior to surgery and repeated twice postoperatively. The stimulus-induced modulation of cortical alpha oscillation changed from pre- to 12 months postoperative recording in simple attention tasks. Neuropsychological examination pointed to improvement in postoperative motor dexterity and working memory. Our tentative results suggest that MEG may provide a useful means to evaluate CEA-related changes in the brain. Additional analyses in larger samples with age-matched controls are required to confirm the link between the changes in brain function and possible improvement in cognitive function in these patients. Increased understanding of the consequences of CEA on brain function and cognition is important both to clinicians and patients.

## Introduction

Atherosclerotic stenoses in the carotid bifurcation or in the proximal part of the internal carotid artery cause 10–16% of all cerebrovascular accidents [1]. Typically, they cause transient or permanent motor dysfunction, speech disorders or visual loss. Symptomatic, hemodynamically significant (50–99%) stenoses can be treated invasively, preferably surgically [2]. Carotid endarterectomy (CEA) is prophylactic in nature; in other words, it is performed to reduce stroke risk in the future. In the operation, the narrowing, often irregular, and therefore prothrombotic plaque is removed. This invasive treatment can be offered for recently symptomatic patients with hemodynamically significant carotid stenosis who have not suffered a major stroke or who show signs of good recovery. CEA carries a certain risk for stroke in itself, but if the risk for stroke is higher without an operation, invasive treatment is justified.

Although the aim of operative treatment is stroke risk reduction, the operation may impact cognitive function as well [3–4]. Discussions on potential changes in cognition following CEA are common with carotid patients and their relatives. Opening of a stenotic artery and restoring blood flow to the brain may improve cognitive function by relieving chronic hypoperfusion [5]. Furthermore, removing atherosclerotic lesions from carotid bifurcation eliminates a potential source of emboli. Those emboli might, in turn, cause new ischemic lesions in the brain. On the other hand, CEA may lead to cognitive decline caused by procedural emboli [6], general anesthesia, temporal carotid flow interruption during clamping or postoperative hyperperfusion [7]. During the last decade or two, advances in procedural techniques and patient care have shown to improve postoperative cognitive functioning in CEA patients [8]. In the literature, cognitive outcomes after CEA are, however, inconsistent [9]: deterioration [10–12], no change [13,14] or improvement have all been reported [3–5,15–22].

Different methods have been used to measure this change in cognition in patients undergoing CEA. Clinical screening tests (MMSE, MoCa) [15,18], specific neuropsychological examinations (NPEs) [22] and comprehensive cognitive evaluations of the impact of CEA on cognition have all been performed [10,23].

Changes in cognitive functions following CEA presumably reflect hemodynamic and electrophysiologic changes in the brain. CEA has been shown to reduce hypoxic but viable brain tissue [19]. Furthermore, carotid revascularization has been associated with increased regional cerebral blood flow and cerebrovascular reactivity in conjunction with the disappearance of theta activity in some patients [24,25]. Regional cerebral blood flow and cerebrovascular reactivity have been measured by transcranial doppler [23], SPECT and PET [19,24], as well as by functional magnetic resonance imaging (MRI) [26]. Functional resting state MRI has also shown increased regional neural activity following CEA [18]. Importantly, changes in cerebral blood flow have been linked to changes in the oscillatory electrophysiological activity in the brain [27].

From the perspective of cognitive competence, electrophysiological measures indicate the efficiency and integrity of information processing in cortical networks. To examine the effects of CEA on the information processing in the brain, electrophysiological brain imaging methods, such as magnetoencephalography (MEG) and electroencephalography (EEG), may provide currently largely unused potential for clinical research and practice. MEG measures the magnetic fields produced by the electrical activity of the brain and enables to compare both the spatial and temporal characteristics of neural activation during rest or cognitive tasks. MEG and EEG can be used to track the engagement of neural resources in task performance with millisecond accuracy. In the context of CEA, electrophysiological measures have been applied to monitor ongoing brain state during the operation (EEG, SEP) [14,16,20], but EEG based P300 evoked potentials have also been measured to assess changes in cognition in the follow-up of CEA patients [4–5]. In one study, MEG recordings during awake eyes closed condition evidenced misery perfusion in the form of temporo-parietal theta activity caused by correctable stenoses in the internal carotid and middle cerebral arteries in some of these patients [24]. However, we are not aware of previous studies that used serial MEG recordings during cognitive load to examine potential changes in mid-term follow-up on symptomatic CEA patients.

Carotid stenoses causing hypoxia are associated with various cognitive dysfunctions, and revascularization surgery has been shown to specifically improve so-called executive functions: working memory, attention and motor speed/efficiency [13,17,22,23,28]. However, there are also studies reporting no changes in these cognitive functions [29,30].

Based on this earlier literature, we hypothesized that operative treatment might ameliorate brain function and associated cognitive functions, specifically in tasks that require focusing of attention, working memory and visuomotor speed. Besides a comprehensive neuropsychological test battery targeted specifically at executive functions, we also measured brain activation during tasks requiring varying levels of attentional control and working memory. Attention and working memory have been widely shown to particularly rely on oscillatory signalling at alpha (and theta) band [31–34]. These robustly accessible markers of brain activity thus offer feasible metric that can reveil possible changes in executive functions in the context of CEA. Therefore, building on previous findings that link alpha and theta rhythms to attention and memory processes [31,35–37], as well as those linking theta modulation to changes in cerebral blood flow [24,25], we focused on these frequency bands as key neural markers of cognitive functioning.

In this pilot study, we aimed at establishing the feasibility of using MEG to detect changes in neuronal activity in patients with recently symptomatic carotid stenosis and CEA. We also tested the changes in cognitive performance profile following CEA. Ultimately, we aim at achieving deeper understanding of the consequences of CEA on brain function and cognitive performance in this patient group.

## Methods

### Study design

**Fig 1:**
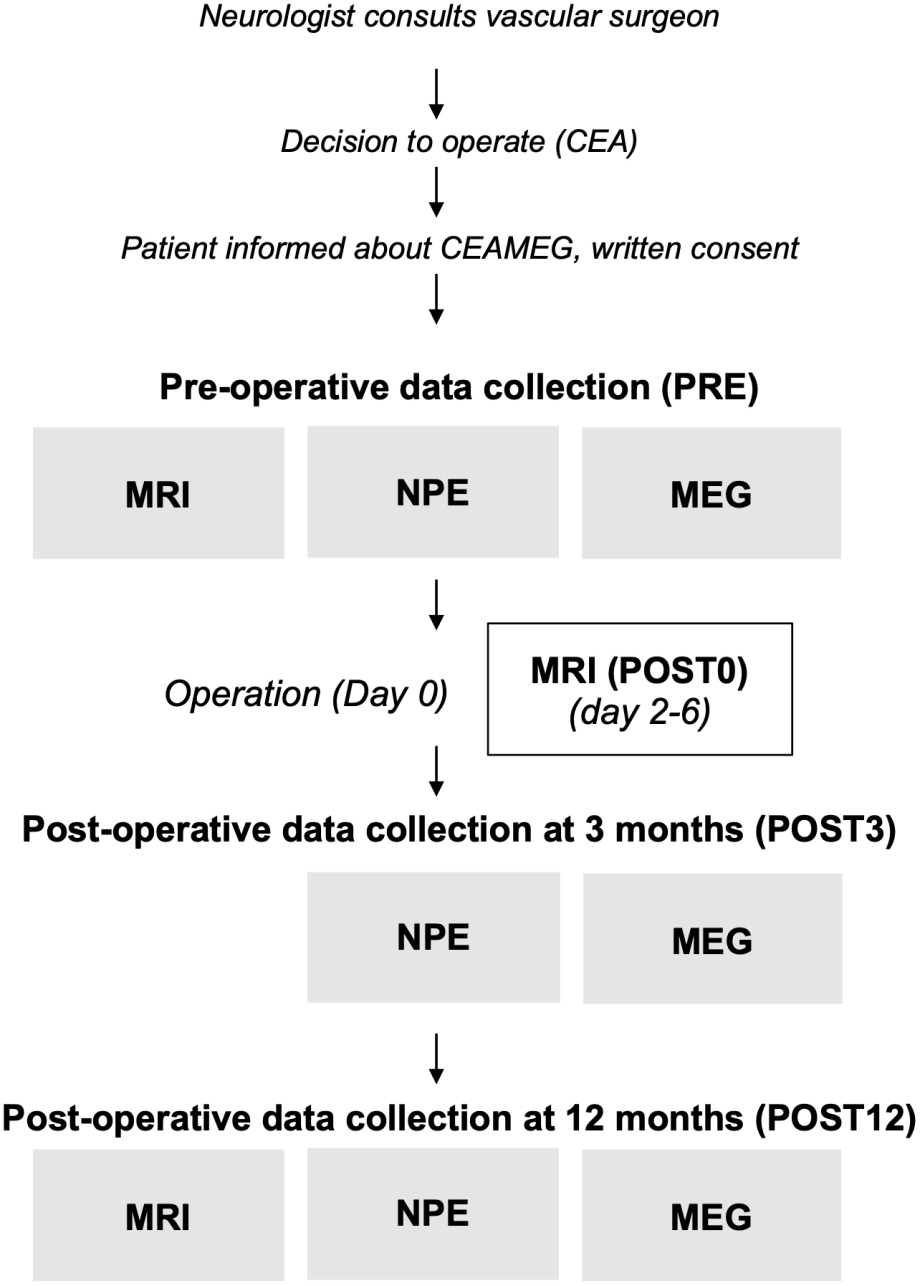
Flowchart of the study.

Recently symptomatic CEA patients were prospectively enrolled in this study. Prior to the surgery, patients underwent head MRI, NPEs and MEG (PRE) during the following days from the decision to operate, since delay from the symptom onset to surgery should preferably be kept to less than 14 days [9]. MRI was repeated postoperatively (MRI POST0) before discharge to detect potential new lesions associated with CEA. Follow-up MEG and NPEs were conducted at 3 months (POST3), and repeated again at 12 months (POST12) supplemented by MRI (Fig 1).

### Participants

Six recently symptomatic CEA patients (Table 1) were prospectively enrolled in this observational study between May 5^th^ 2017 and August 30^th^ 2018. Patients could be included if they had recent symptoms due to a hemodynamically significant (>50%) carotid stenosis and had been scheduled for a CEA. The exclusion criteria were severe stroke (modified Rankin score >3) [38], crescendo TIA, stroke-in-evolution, marked cognitive impairment, substance dependence, active malignant disease, marked psychiatric disorders, medication affecting the central nervous system, lack of co-operation and metal foreign bodies inhibiting MRI. Unfortunately, one of the six patients experienced a major perioperative stroke, which led to the exclusion of that patient. Therefore, the analyses comprised of five patients.

**Table 1:** Patient characteristics and operative details.

Characteristic	Values
**Number of patients**	5
**Age** (Mean, median, range)	73, 72, 69–77 yrs.
**Sex**	
Female	1/5
Male	4/5
**Operative indication**	
Minor stroke	1/5
AFX	1/5
Transient ischemic attack (TIA)	3/5
**Risk factors**	
DM II	2/5
Hypertension	4/5
Coronary artery disease	3/5
Atrial fibrillation under treatment	2/5
Smoking	2/5
**Lesion characteristics**	
Stenosis % (NASCET) (mean, median, range)	72, 70, 55–90%
Side (right and left)	2 and 3/5
Contralateral carotid stenosis (>50%)	2/5
Vertebral artery stenosis (>50%)	2/5 (bilateral in one patient)
**Delay**	(Mean, median, range)
From index event to neurologic evaluation	12, 0, 0–50 days
From neurologic evaluation to decision to operate	5, 1, 0–20 days
From decision to operate to surgery	10, 8, 8–12 days
From index event to surgery	27, 13, 9–79 days
**Preoperative medication**	
Statin	5/5
ASA^1^ + Clopidogrel	3/5
Dipyridamol + ASA^1^	1/5
LMWH^2^	3/5
**Operative details**	
Locoregional anesthesia	4/5
Patch	5/5
Shunt	1/5

^1^Acetylsalicylic acid.

^2^Low molecular weight heparin.

Although CEA is performed to reduce future stroke risk, the operation carries a certain risk for stroke in itself. According to recent guidelines, the in-hospital stroke and death rate for symptomatic CEA patients should be < 4%, which corresponds to the more traditionally reported 30-day threshold of < 6% [2]. The 30-day disabling stroke and death rate in the 5-year period at this institution was 1,3% at the time of the study.

### Ethics approval and consent to participate

Approval of the ethical committee of the Central Finland Health Care District (7U/2016) was obtained before the start of the study. Written informed consent was obtained from all patients. The methodology in this study was in accordance with the relevant guidelines and regulations.

### Magnetic resonance imaging

Serial magnetic resonance imaging (MRI) of the brain (1.5 T Signa, GE Healthcare, Chicago, Illinois, USA or Symphony, Siemens, Erlangen, Germany) was used to document acute or more chronic brain infarctions, other structural abnormalities, white matter hyperintensities (FAZEKAS rating scale) signaling chronic small vessel ischemia and the degree of medial temporal lobe (Hippocampal) atrophy (SCHELTENS rating scale) during the course of the study (Fig 1). The following sequences were used: Anatomic 3D T1, 2 D T2, Flair and diffusion-weighted imaging (DWI). The scans were rated in a blinded manner by an experienced neuroradiologist. A summary of the serial MRI findings is displayed in Table 2.

**Table 2:** Findings of serial MRIs.

	Patient 1	Patient 2	Patient 3	Patient 4	Patient 5
**Operative indication** **(side of the CEA), other notes**	TIA (right)	Minor stroke (left), (right carotid occlusion)	TIA (left)	TIA (left)	AFX (right), old (20 yrs) ipsilateral stroke, contralateral stenosis 90%
**Preoperative signs of ischemia**	None	Acute bilateral FPO ischemia in MRI PRE turning to subacute ischemia in MRI POST0	None	Acute left F (Broca) ischemia	None
**Perioperative ischemic findings**	Acute FP ischemia (right) in MRI POST0, disappeared by MRI POST12	None	None	None	None
**Chronic ischemia**	C (capsula externa, right) in all MRIs	C (corona radiata, right) in all MRIs	No	No	F (right) in all MRIs
**Progressing atrophy (GCA)**	T and Ce, progression 1 > 2 from MRI POST0 to MRI POST12	FPCe, progression 1 > 2 from MRI POST0 to MRI POST12	Present all the time, no progression	Present all the time, no progression	Present all the time, no progression
**FAZEKAS**	3 (FPO)	1 (FP)	3 (FPO)	3 (FP)	1 (FP)
**Micro-** **hemorrhages**	None	Bilateral PO in MRI PRE and POST0, bilateral P in MRI POST12	4 small hypertensive/amyloid microhemorrhages in MRIs PRE and POST0 turning to potential amyloid angiopathy in MRI POST12	SWI missing in MRI PRE, peripheral amyloid angiopathy in MRIs POST0 and POST3	1 (FP) throughout the study

CEA = Carotid endarterectomy.

TIA = Transient ischemic attack.

AFX = Amaurosis fugax (transient visual loss).

GCA = Global Cortical Atrophy Scale (0–3).

F = Frontal.

T = Temporal.

P = Parietal.

O = Occipital.

C = Central.

Ce = Cerebellar.

FAZEKAS = scale (0–3) for white matter lesions (chronic small vessel ischemia) and deep white matter (DWM) lesions associated with dementia, this rating remained unchanged throughout the study in all subjects.

### Neuropsychological measures

The participants were measured with an extensive neuropsychological test battery to evaluate the general level of linguistic and nonlinguistic reasoning and memory and to explore the impact of CEA on executive functions and motor performance. More specifically, these tests evaluate the visuo-constructive abilities, language comprehension, working memory, verbal and visual learning and recall, sustained attention and processing speed, semantic and phonemic retrieval and motor dexterity of the participants. From the Brief Repeatable Battery of Neuropsychological Tests (BRBNT), two subtests were used (10/36 Spatial Recall, Symbol Digits Modalities Test) [39], and the PASAT-B version with 3” and 2” stimulus intervals [40]. The other tests used include Mini-Mental State Examination (MMSE) [41], Block Design, Similarities and Digit Span from the WAIS-IV test battery Finnish version, Word list I and II from the WMS-III test battery Finnish version [42,43], TMT-b Finnish version [44], Word List Generation and Tapping.

### Clinical procedure

Patients were operated by longitudinal carotid bifurcation arteriotomy, endarterectomy and patch angioplasty. Operative details are presented in Table 1. CEAs were usually performed under ultrasound-guided locoregional anesthesia, which allows awake neurological monitoring throughout the operation. In addition, near infrared spectroscopy (NIRS) monitoring was used to monitor intraoperative cerebral oxygenation [45]. Routine patching with a bovine pericardium patch was used to close the arteriotomy, while shunting was selective. Postoperative hyperperfusion syndrome was prevented by active control of blood pressure following the restoration of carotid flow. One patient was excluded due to a perioperative major stroke, causing inability to participate in the subsequent follow-up investigations. Therefore, the follow-up analyses comprised five patients.

### Magnetoencephalography

#### MEG data acquisition.

Brain activity during visual cognitive tasks was recorded using a 306-channel whole-head Elekta TRIUX MEG system (Megin Oy, Helsinki, Finland) in a magnetically shielded room (Vacuumschmelze GmbH, Hanau, Germany). The MEG signals were acquired at a 1000 Hz sampling rate and band-pass filtered at 0.03–330 Hz online. Prior to the measurement, five head position indicator (HPI) coils were attached to the subject’s head (three in the front and two behind both ears). A 3-D digitizer was used to determine the coil locations in reference to three anatomical landmarks (nasion and bilateral preauricular points). Two electro-oculogram (EOG) electrodes were attached above the right eye and below the left eye to monitor eye movements and blinks. An electrocardiogram (ECG) was recorded with two electrodes. Eye movements and cardiac activity give rise to strong signals in MEG sensors; therefore, it is important to remove those artifacts prior to further analyses. During MEG data acquisition, the subjects were comfortably seated under the MEG helmet-shaped sensor array. They were instructed to avoid excessive movements and to try to relax and focus on the tasks. A response pad was positioned on a table in front of them, with response buttons indicating “yes” and “no” answers as appropriate. Before starting the data collection, the position of HPIs with respect to the MEG sensors were defined allowing continuous tracking of the head movements during data acquisition [46]. The locations of HPIs were used in the MEG preprocessing stage to make corrections for head movements and to co-align the head position between the tasks to enable channel-to-channel comparisons.

#### MEG measurement protocols.

We collected resting state data (8 minutes eyes closed and 4 minutes eyes open) to evaluate the ongoing dynamics of electrophysiological brain activity at rest. The active part of the protocol consisted of three separate visual tasks: 1) *Simple attention task*, where subjects were required to follow a stream of numbers and press a button whenever the number was “1”; 2) *Simple working-memory task*, where the subjects were presented with a stream of letters, and when a question mark appeared, they were required to report the letter shown 2 letters earlier (2-back task); 3) *Complex attention/working-memory task*, modified from PASAT-B, where the subjects were presented with a stream of numbers, and they were asked to keep count of the sum of the two consecutive numbers and to respond if the sum was the number shown or not whenever a square appeared around a shown number. In all tasks, the stimuli appeared with 2700 + −250 ms interstimulus interval (ISI), and stimulus duration was 300 ms (except for the first two sessions of the first subject, ISI and stimulus durations were 800 ms in task 2). All subjects completed all tasks in every session. Due to the small number of successful trials in complex attention/working -memory task, we analyzed only the data of the simple attention and working memory tasks.

#### MEG data analysis.

**Preprocessing.** The raw data were first preprocessed using Maxfilter v.2.2 software (Megin Oy, Helsinki, Finland). The spatiotemporal signal space separation method (tSSS) [47] was used to remove external and patient-related (e.g., braces, stitches) magnetic interference, detect bad measurement channels, co-align head positions to that of the complex task, compensate for head movements, and downsample the data by a factor of 3. The magnetic artifacts generated by cardiac and ocular activity were then removed using an independent component analysis (ICA). The eliminated components were manually identified based on visual inspection of the field topography and by comparing their time courses to the recorded EOG and ECG time courses.

**Time–frequency analysis.** The data analysis was conducted with custom scripts utilizing the MNE-Python software [48], following the standard analysis pipeline for MEG time–frequency representations (TFRs). We included only gradiometer channels in the analysis, as they show the maximum signal right above the active cortical area and therefore offer an easier interpretation of the spatial distribution of activity. First, we extracted the epochs in each channel for the visual stimuli separately in the two conditions: 0-back and 2-back tasks (Table 3). We used an epoching window of –1500–2500 ms, with respect to the stimulus onset and a rejection limit of 6e-10 ft/cm. Epochs were baseline corrected from –0.5 to 0 seconds before transformation into TFRs. The average evoked response over all epochs was subtracted from the epoch time courses to remove the effect of the phase-locked signal from the resulting TFRs. TFRs were calculated with a Morlet wavelet transformation [49], in a frequency range between 2 and 35 Hz with 17 wavelet center frequencies evenly distributed on a logarithmic scale, and the number of cycles in the Morlet wavelets was equal to half of the center frequency value (in Hz). Average TFRs were calculated for sensors in nine scalp areas: left and right frontal, temporal, parietal and visual areas as well as the vertex. Channel group averages were taken across TFR-epochs grouped by condition (measurement, session idx, subject id, frequency bin and time for TFR heatmap plots and by measurement, session idx, subject id, band and time for TSE (temporal–spectral evolution) plots. Mean TFRs were rescaled as follows, using –1.0 to 0 s as the baseline to compute the scaling for each center frequency: dividing by the mean of baseline values, taking the log, and dividing by the standard deviation of the log of baseline values (“zlogratio”). This rescaling helps to assess the significance and robustness of the changes and makes them more comparable between subjects.

**Table 3:** Number of epochs after artefact removal across subjects by session and tasks.

	n of accepted trials		
Subject	PRE (0-back/2-back)	POST3 (0-back/2-back)	POST12 (0-back/2-back)
1	132/150	180/150	132/159
2	131/174	145/160	153/157
3	149/154	149/155	151/164
4	150/165	133/136	153/163
5	148/172	144/148	151/151
% of accepted out of all epochs	99,2/98,8	99,5/98,9	98,8/99,0

Based on earlier literature, we focused our analysis on alpha band oscillatory activity between 8–12.0 Hz and theta band at 3–7.0 Hz [33,34]. To examine the time-varying changes in the oscillatory power in these frequency bands, we computed the TSE plots [50]. The TSE time courses were extracted separately for the attention (0-back) and working memory (2-back) conditions, thus reflecting the changes in alpha power induced by these different tasks.

The influence of CEA on neural engagement during the tasks was evaluated by comparing the TSE time courses at alpha and theta bands before CEA (PRE) with the 3 months after CEA (POST3) and 12 months after CEA (POST12). This evaluation was done both visually case by case and by statistical analysis.

### Statistical testing

Due to the small number of subjects, we report the results first at the individual subject level for both cognitive performance and MEG data. In addition, we perform a tentative statistical analysis to provide a preliminary estimate of the effect size and direction at the group level, with the aim of illustrating potential trends rather than making strong inferential claims. For neuropsychological variables, repeated measures analysis of variance (rANOVA) was used to evaluate the effects of time (PRE, POST3 and POST12) on cognitive performance. On standardized tests, the cutting points were used according to the test manuals (subtests of WAIS-IV and TMT-B). The sphericity of the models was checked.

For MEG signals, the post-stimulus period (0–2000 ms) was divided into four equal 500-ms bins to obtain stable estimates of alpha and theta power while capturing their temporal dynamics. We extracted power values (area under curve) in these time-windows (TW) of the TSE curves: 0–500 ms (TW1), 500–1000 ms (TW2), 1000–1500 ms (TW3) and 1500–2000 ms (TW4), separately for each individual for alpha and theta band of the TSEs, and separately for the two task conditions (“attention” and “working memory”) and for each area of interest. TSE confidence intervals were calculated by subtracting or adding the standard error of the mean across epochs multiplied by 1.96 to the mean, and rescaled with the same parameters as the mean across epochs. rANOVA was used to evaluate the effect of session (PRE, POST3 and POST12) on the power of alpha oscillation in 0–500 (TW1), 500–1000 (TW2), 1000–1500 (TW3) and 1500–2000 (TW4) ms time-windows. The influence of the hemisphere was also tested by including the interaction between the session and the hemisphere. Thus, a 3 (session) x 2 (hemispheres) rANOVA was performed on each TW. In the case of a significant main effect, simple contrast PRE vs. POST3 and PRE vs. POST12 were used to test separately the short- (PRE vs. POST3) and long-term (PRE vs. POST12) effects. Statistical analyses were restricted to regions for which time courses could be reliably extracted for all participants (5/5) ensuring complete data coverage; these regions were occipital and temporal areas.

## Results

### Cognitive performance

Tapping speed increased on average across subjects from 42 taps to 47/10 seconds in the right and from 38 taps to 43/10 seconds in the left hand when comparing the results at 12 months after the operation with the preoperative ones. Furthermore, digit span standard scores increased on the average by 3 steps in each patient. There was a main effect of time in Tapping right hand (F(2,8)=4.56, p < 0.05), Tapping left hand (F(2,8)=6.03, p < 0.05) and Digit Span (F(2,8)=8.07, p < 0.05. The tests of within-subject contrasts showed that the performance in the last measuring point (POST12) improved from that of the first measuring point in all the aforementioned tests: Tapping right hand (F(1,4)=2.67, p < 0.05), Tapping left hand (F(1,4)=16.09, p < 0.05) and Digit Span (F(1,4)=15.47, p < 0.05). This indicates that the dexterity of both hands of the patients as well as working memory improved at 12 months postoperatively compared with the preoperative situation. In TMT-b, the main effect of time was close to significant (F(2,8)=4.40, p = 0,051). There were no statistically significant changes in other neuropsychological measures. The results of cognitive testing are presented in Table 4.

**Table 4:** Cognitive test results.

Test	Preoperative	Postoperative 3 months	Postoperative 12 months	Time	PRE vs. POST3	PRE vs. POST12
Tapping right hand	41.93 (3.99) [37.33-46]	45.20 (2.97) [40.67-49]	47.06 (6.35) [39.33-53.30]	0.048*	0.059	0.024*
Tapping left hand	38 (4.79) [31-42.67]	41.60 (6.23) [32.33-49.67]	43.33 (7.45) [31.33-50.33]	0.025*	0.108	0.016*
MMSE	26.80 (3.96) [20-30]	26.20 (1.30) [25-28]	27.20 (2.28) [24-30]	0.738	0.710	0.740
Word List Generation	21.60 (3.36) [18-27]	23.60 (6.27) [17-30]	24.80 (5.22) [20-32]	0.298	0.351	0.199
Spatial Recall	16.20 (6.3) [9-25]	19.20 (4.76) [15-26]	18 (5.34) [13-27]	0.326	0.262	0.387
Block Design	24.20 (9.91) [10-34]	20.60 (8.65) [10-33]	25.40 (6.91) [19-36]	0.428	0.374	0.654
Similarities	26.20 (2.28) [23-29]	26.20 (2.49) [24-30]	26 (3.08) [22-29]	0.769	0.501	0.596
Digit Span	20 (3.32) [17-24]	22.20 (3.63) [18-28]	24.20 (2.28) [22-28]	0.012*	0.086	0.017*
SDMT	24.20 (9.94) [9-33]	19.40 (9.84) [8-32]	28.20 (13.07) [13-46]	0.396	0.440	0.331
Word List Generation, delayed	3.60 (2.88) [0-8]	3.60 (2.41) [1-7]	3.80 (1.92) [2-7]	0.922	1.0	0.704
Spatial Recall, Delayed	5.80 (1.92) [4-9]	6 (3.32) [1-9]	5.4 (1.67) [4-8]	0.808	0.847	0.374
PASAT 3”	25.20 (3.83) [20-30]	24.75 (5.12) [19-30] n = 4	24.80 (6.26) [19-34]	0.972	1.0	0.848
PASAT 2”	17.80 (8.93) [9-30]	22.75 (7.81) [13-30] n = 4	20.40 (9.29) [9-30]	0.718	0.320	0.832
Word Fluency (Animals)	17.40 (5.68) [9-24]	20 (5.83) [14-28]	19.20 (5.07) [13-27]	0.488	0.256	0.494
Word Fluency (s)	11.20 (5.40) [5-18]	13 (3.67) [8-17]	11.80 (4.21) [9-19]	0.706	0.529	0.710
Word Fluency (k)	13.20 (2.59) [9-16]	15.80 (4.60) [9-19]	15.60 (4.16) [11-20]	0.105	0.081	0.109
TMT (b)	225.40 (81.94) [116-300]	155.40 (26.83) [121-188]	157.60 (54.32) [100-239]	0.051	0.075	0.079

Presented values are: mean, (standard deviation), [range].

Values express correct answers except in tapping (taps in 10 seconds), similarities and block design (standardized scoring values) and TMT-B (seconds). P values for time, preoperative vs. postoperative 3 months and preoperative vs. 12 months.

* = statistically significant change (p < 0.05).

### Magnetoencephalography: induced changes in oscillatory activity at alpha and theta bands

**Fig 2:**
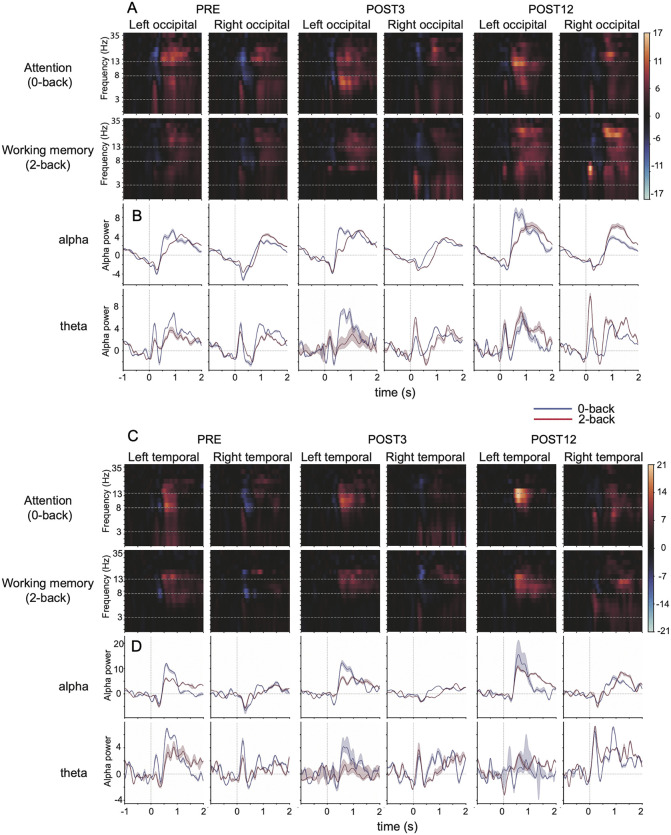
Time–frequency representations (TFR) and band-passed filtered oscillatory power (i.e., temporal–spectral evolution or TSE) at alpha and theta bands in one subject. **a)** TFR for PRE, POST3 and POST12 sessions in occipital area for attention condition (upper row) and working memory condition (lower row). **b)** TSE time-courses for alpha (upper row) and theta (lower row) modulation in PRE, POST3 and POST12 sessions in occipital area. **c)** TFR for PRE, POST3 and POST12 sessions in temporal area for attention condition (upper row) and working memory condition (lower row). **d)** TSE time-courses for alpha (upper row) and theta (lower row) modulation in PRE, POST3 and POST12 sessions in temporal area.

Modulation of alpha oscillation followed the typical behavior reported in the earlier literature on visual attention and visual working memory tasks (Fig 2 and S1 Fig). First, there was a decrease in the level of alpha oscillation shortly after stimulus presentation, most clearly evident in occipital areas and visible in 5/5 participants. This decrease in alpha oscillation (in reference to the baseline period) lasted until about 1000 ms, and it was followed, and partly overlapped, by an increase in the level of alpha oscillation. The increase in alpha oscillation was also visible in 5/5 individuals but showed a somewhat more variable pattern in spatial distribution. In most of the individuals, it was evident in the occipital area but could also be seen in temporal areas (in 5/5 individuals) and occasionally in the parietal (3/5) and frontal areas (3/5).

The distribution of oscillatory activity during the working-memory task, as evidenced by the TFR plots (Figs 2 a and c) and TSE time courses (Figs 2 b and d), demonstrates notable within-individual replicability across time. In other words, while individuals exhibit clear differences in the task-related distribution of oscillatory activity, their characteristic alpha patterns remain highly consistent across repeated measurements, even when recorded 12 months apart (S1 Fig pages 1 and 2).

In subject-by-subject examinations, specifically, the increase in alpha oscillation indicated possible changes between pre- and postoperative measurements. By visual inspection, in 4/5 individuals, the level of alpha increase after stimulus presentation was enhanced in the postoperative measurements, but this enhancement was more often evident for the 0-back task (and not for 2-back) (S1 Fig).

### Magnetoencephalography: The effect of CEA on oscillatory modulation during attention and working memory tasks

For those areas where TFR indicated a modulation in oscillatory activity induced by the stimulus presentation in all subjects (i.e., both right and left occipital and temporal areas), the effect of CEA on task-induced oscillatory power in short- and long-term follow-up was tested by rANOVA. Based on earlier literature and the frequency distribution in current data (Fig 2 and S1 Fig), the analysis was conducted for alpha and theta bands.

For the simple attention condition, measurement session (i.e., PRE, POST3 and POST12) showed a significant main effect for the alpha power at 500–1000 ms (time-window 2) after stimulation similarly in the left and right temporal areas (F(2,8)=8.6, p < 0.01, η²p = 0.68). Note that the main effect for hemisphere was significant (F(1,4)=9.5, p < 0.05, ηp² = 0.70), but the hemisphere×session interaction was not (F(2,8)=0.28, p = 0.77, ηp² = 0.064)). Given the small sample (n = 5), effect size estimates should be interpreted with caution. In post hoc tests based on estimated marginal means (Bonferroni-adjusted), the difference was significant between POST12 vs. PRE (p = 0.043, mean change= + 1.149 arbitrary units [a.u.], 95% CI [0.048, 2.250]), but not between POST3 vs. PRE (p = 1.000, mean change= + 0.161 a.u., 95% CI [−0.939, 1.261]) or POST12 vs. POST3 (p = 0.133, mean change= + 0.988 a.u., 95% CI [−0.365, 2.342]). There were no significant effects in alpha power in the occipital area, nor any effects in the temporal or occipital area for theta power.

**Fig 3:**
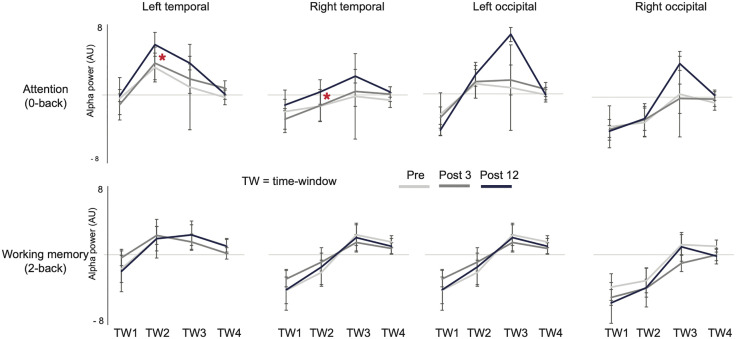
The average alpha power in four time-windows (0-500, 500-1000, 1000-1500 and 1500-2000 ms) in the occipital and temporal areas across PRE, POST3 and POST12 measurement sessions for the working memory and attention condition. A significant main effect of session in TW2 from 3 (session) x 2 (hemisphere) repeated-measures ANOVA is indicated by red asterix.

For the working memory condition there were no differences in oscillatory power between measurement sessions, either in the temporal or occipital area, and either for alpha or theta power. Fig 3 depicts the alpha power values in each time-window after stimulation, both for temporal and occipital areas, and illustrates the changes from PRE to POST3 and POST12 measurement sessions for both task conditions.

## Discussion

We explored the feasibility of using task-related MEG recordings, together with a neuropsychological test battery, to identify potential changes in the neurocognitive status of symptomatic CEA patients. Brain electrophysiology and cognitive performance were evaluated at 3 and 12 months after CEA, and contrasted with the preoperative state. The comprehensive test protocol (NPE, MRI, MEG) appeared to be feasible to perform on recently symptomatic carotid patients, taking into account institutional resources, the status of the patients and the recommendations to perform CEA within 2 weeks of the ischemic event. While none of the cognitive measures indicated deterioration in pre–post comparison, performance in fine motor skills and working memory improved in this small patient group. In case-by-case visual analysis, MEG paradigms probing the neural basis of simple working memory and attention demonstrated the modulation of oscillatory power at the 10 Hz alpha band during tasks of attention and encoding of visual information, which is in line with earlier findings in non-clinical samples [32,33,35–37]. The indicative statistical comparison suggested posttreatment changes in alpha engagement during attentional, but not working memory task, in the long-term (12 months) follow-up.

Following an ischemic event (i.e., clinical symptoms of TIA or stroke), patients with significant carotid stenoses should be diagnosed and scheduled for CEA in less than 2 weeks, since the risk for a new cerebrovascular event remains high despite optimal medical treatment [2,9,51]. Analogously, the earlier the patients are operated on, the greater the proportion of them who will benefit from the surgery. The same applies to patients taking part in research projects. None of the operations were postponed due to the decision to participate in the study. Our 5-case pilot cohort of CEA patients showed that it was feasible to organize MRI, NPEs and MEG preoperatively within tight timeframes. Investigations included in the study protocol were well tolerated by the patients, despite their recent cerebrovascular incidents. In the MEG recordings, resting state and simple attention/working-memory tasks were successfully performed by all participants, but the most challenging cognitive task with high demands for working memory and attention, appeared to be too difficult for this study population.

CEA is performed to diminish stroke risk in patients with either symptomatic or asymptomatic hemodynamically significant carotid stenosis. However, cognition even in asymptomatic carotid patients with severe stenoses before any invasive treatments has been found compromised when compared with a matched population-based cohort [52]. Whether CEA affects postoperative cognition or not, and if so, how and why, remains controversial [9]. The authors of a recent systematic review suggested, that some of the asymptomatic carotid patients with impaired cerebral hemodynamics and cognitive impairment might actually be regarded as symptomatic after all [53]. Currently, there is no evidence to support CEA in asymptomatic carotid patients to prevent or reverse cognitive deterioration [54].

In this pilot study, the focus was on recently symptomatic carotid patients. The neuropsychological examinations showed no evidence of performance decline in a 12-month follow-up when compared to the preoperative situation. On the contrary, working memory and motor dexterity, both in the right and left hands, showed improvement at 12 months. Analyses on MEG recordigs suggest improvement in attention. In general, improvements in working memory, attention and motor functions are well in line with earlier studies on cognitive outcome and CEA [17,22,23,55]. Some studies have, however, reported either improvements in other cognitive functions, such as verbal fluency and visual perception [56], or no effects [29]. Differences on the cognitive outcome partly reflect the variability in the cognitive tests used [5]. Also, in some studies, a cognitive sum score has been reported [29]. The timing of the follow-up examinations varies across studies, making it difficult to reliably compare the findings. Also, the date of the operation itself matters. During the decades, CEA has become more advantageous, what comes to improvement in cognition [8]. Furthermore, many studies include heterogenous populations of patients with symptomatic and asymptomatic patients [17,23,55] or combine outcomes of carotid stenting and CEA [5,12]. Although our small sample does not allow us to draw firm conclusions about changes in cognitive functions, the improvement in performance measures in each individual aligns with those earlier studies that indicated changes particularly in working memory and motor functions. It is noteworthy, that although in some of the patients in the current study cortical atrophy increased during the follow-up, as could be expected in this age group, cognitive functions did not deteriorate.

In the current literature, there is no consensus of the most sensitive cognitive domain for the positive effects of improved brain perfusion following CEA. However, the contemporary findings, including the present results, seem to show the most systematic changes in those cognitive functions that require a high level of arousal, as well as speed and efficiency of information processing. In general, the cognitive functions that typically change following CEA seem to be the same that react sensitively to hypoxia also in other contexts [57]. It is difficult to rule out other factors that possibly influence the cognition following CEA, such as the age [12], individual variance in cognitive or brain “reserve” [58] and the preoperative extent and location of the hypoxic brain tissue [19]. Moreover, it is possible that not all brain networks, and consequently not all cognitive functions, are equally influenced by changes in perfusion-dependent tissue oxygenation. Cortical neurons show evidence of the capability to adjust their performance based on available nutrients [59]. More specifically, food restriction in mice resulted in an increase in response variability and a decrease in visual tuning, suggesting that neurons can downregulate their level of activity in response to a shortage of vital compounds. Therefore, it may turn out to be central to pinpoint which cognitive domains are prone to display improvements following reperfusion after CEA. Most certainly, not all cognitive functions are equally capable to improve at older age, not even after successful revascularization. Issues on the effect of CEA on cognition emerge in clinical practice, and it is therefore important to increase understanding of the implications of CEA in symptomatic carotid patients.

MEG provides millisecond-accurate information on neuronal information processing, which underlies cognitive functions in the intact and defective human brain. Besides researching the brain basis of cognitive functions, MEG provides individually meaningful information that has proven useful for diagnostics in specific clinical situations. In current clinical practice, MEG is used mainly in the diagnostics of epilepsy, particularly for presurgical localization of epileptic activity [60]. However, it is also employed to guide surgical operations ensuring that critical areas for cognitive functions, such as speech and hand movements, are preserved [61]. This exemplifies the technique’s sensitivity in identifying brain processes that underlie functional and non-functional brain networks. Indeed, this capability allows for various applications that leverage either resting state dynamics or task-related patterns in brain electrophysiology to identify potential imaging biomarkers for mild traumatic brain injury [62], early-stage Alzheimer’s disease [63], prognosis in multiple sclerosis [64], or autism spectrum disorders [65].

To advance our understanding of the possible implications of CEA outside current clinical indications, modern neuroimaging tools may provide essential information. MEG is applicable for detecting the functional consequences in the brain caused by carotid revascularization, subsequent increase in brain perfusion and positive changes in tissue oxygenation [24]. Unilateral temporoparietal theta rhythm has been shown to correlate with ipsilateral carotid occlusive disease in awake state in subsets of these patients [25]. In this pilot study with a small number of subjects, CEA appeared to systematically modulate the oscillatory activation associated with visual attention. The cognitive changes in working-memory performance were not accompanied by changes in oscillatory dynamics during the working-memory N-back task. This is somewhat unexpected, as the more demanding working-memory tasks could be assumed to show more robust effects.

We are not aware of any published reports on CEA-related MEG-changes in task-related neuromagnetic activity. Given the small number of subjects, our results should be regarded as preliminary. The replicable spectrotemporal distribution of task-induced oscillatory dynamics across sessions in each individual supports the interpretation of changes in alpha power. In other words, the same task elicited a comparable pattern of activity across measurement sessions in a particular patient. However, for some parts, alpha-power showed significant positive differences in PRE vs. POST12 measurements.

It is interesting that both the improved cognitive functions and the changes in alpha power in MEG were only observed in the long-term follow-up and not in the 3 months follow-up. This result is supported by earlier findings [15–17] and suggests that the possible neurocognitive recovery of functions after cerebral revascularization takes several months. Although age effects cannot be ruled out in the present study, the improvement, rather than deterioration, of some neuropsychological test measures speaks for CEA-related recovery of neural tissue as underlying the observed findings. Indeed, earlier studies have reported a deterioration of cognitive function in some patients in the early postoperative period (≤ 1 month) following carotid revascularization, most likely reflecting subtle cerebral injuries even in the absence of overt strokes [66], potentially caused by microembolization [6] and operation-related hypo- or hyperperfusion [67]. Of note is the fact that cognitive dysfunction at 3 months after CEA has been associated with increased long-term mortality [68].

In the existing studies on the cognitive outcome of CEA, the timing of postoperative assessment, in addition to a specific pattern of cognitive tests, varies considerably. In our study, the cognitive functions as well as brain effects were first evaluated at 3 months postoperatively, and in this short-term follow-up, cognitive functions did not show evidence of change in either direction. Perhaps the changes that occur in the hypoxic state of the cortex following an ischemic event and subsequent CEA follow a course of initial deterioration followed by improvement only during a longer follow-up. Indeed, temporary changes due to the operation may significantly influence the evaluation of long-term effects [69]. Carefully designed studies with appropriate follow-up times are thus pivotal in determining the ultimate effect of CEA.

Together with the above earlier findings, our results propose a potential benefit of MEG in evaluating the neurocognitive consequences of CEA in symptomatic carotid patients. However, the sample size is small and the results of the indicative statistical analysis are too premature to be generalized to larger populations. Accordingly, the reported effect size should be viewed as preliminary, as effect size estimates are inherently uncertain in small samples. Crucially, in addition to larger sample sizes, it would be important to control patient-related as well as evaluator/clinician-related confounding factors in the future. In addition, the control of potential age-related changes and the possible learning effect in cognitive evaluation has to be taken into account. Unfortunately, parallel versions for some of the cognitive tests were not available in native language for the participants. However, previous studies have demonstrated that all parallel test forms may not be equivalent [70]. Furthermore, it is unlikely the findings obtained in working memory and motor dexterity would reflect learning effects, specifically with the maximum delay of nine months between the measurements. In this study, we did not see improvement on any of the domains usually sensitive for practice effect in older healthy persons and in individuals with mild cognitive impairment (language, executive functions, learning, memory, attention and speed) [70]. Another way to avoid practice effect would be prebaseline massed practice, which would unfortunately be impossible to execute in this patient population within the timeframes in hand. Therefore, in the future, it would be important to have a well matched control group in addition to a larger sample size.

## Conclusions

We tested the feasibility of using combined MEG and neuropsychological test battery to detect changes in neurocognitive status in symptomatic CEA patients during the first postoperative year. The study protocol proved to be feasible with minor modifications and it was well tolerated by the participants. In this small cohort, we observed enhanced postoperative task-induced oscillatory alpha power at 12 months, but not at 3 months. Each patient showed also increase in standardized scores for working memory performance and improvement in manual dexterity. Extending the earlier findings on resting state, this data provides the first evidence of the possible benefit of MEG-derived neural markers during cognitive load in evaluating CEA outcomes. However, additional analyses in larger samples, and across frequency bands, with age-matched controls are required in the future.

## Supporting information

S1 Fig Individual TFR maps and TSE time-courses for alpha and theta modulation for attention and working memory conditions. Page 1: TFR maps for individual subjects at PRE, POST3 and POST12 sessions in occipital areas for attention and working memory condition. Page 2: TFR maps for individual subjects at PRE, POST3 and POST12 sessions in temporal areas for attention and working memory condition. Page 3: TSE time-courses for alpha and theta modulation in individual subjects at PRE, POST3 and POST12 sessions in occipital area, overlaid for attention and working memory conditions. Page 4: TSE time-courses for alpha and theta modulation in individual subjects at PRE, POST3 and POST12 sessions in temporal area, overlaid for attention and working memory conditions.
(PDF)
