# Supplementary figures and images for: Feasibility of MEG in assessing task-related oscillatory markers and evaluating cognitive changes in recently symptomatic carotid endarterectomy patients: A pilot study
Source: PLoS One. 2026 Mar 6;21(3):e0343689. doi: 10.1371/journal.pone.0343689 (PMC12965589; doi:10.1371/journal.pone.0343689)

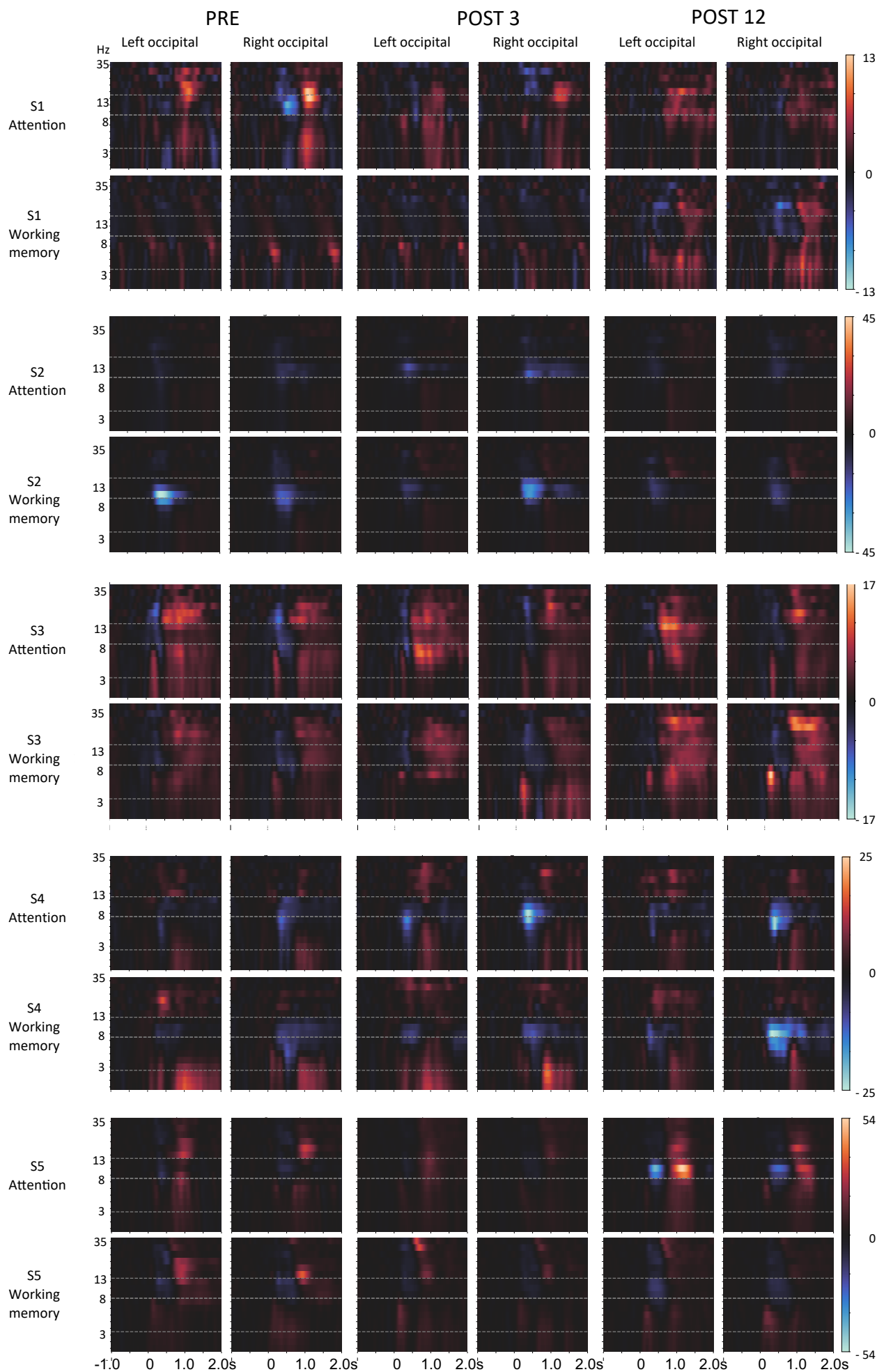

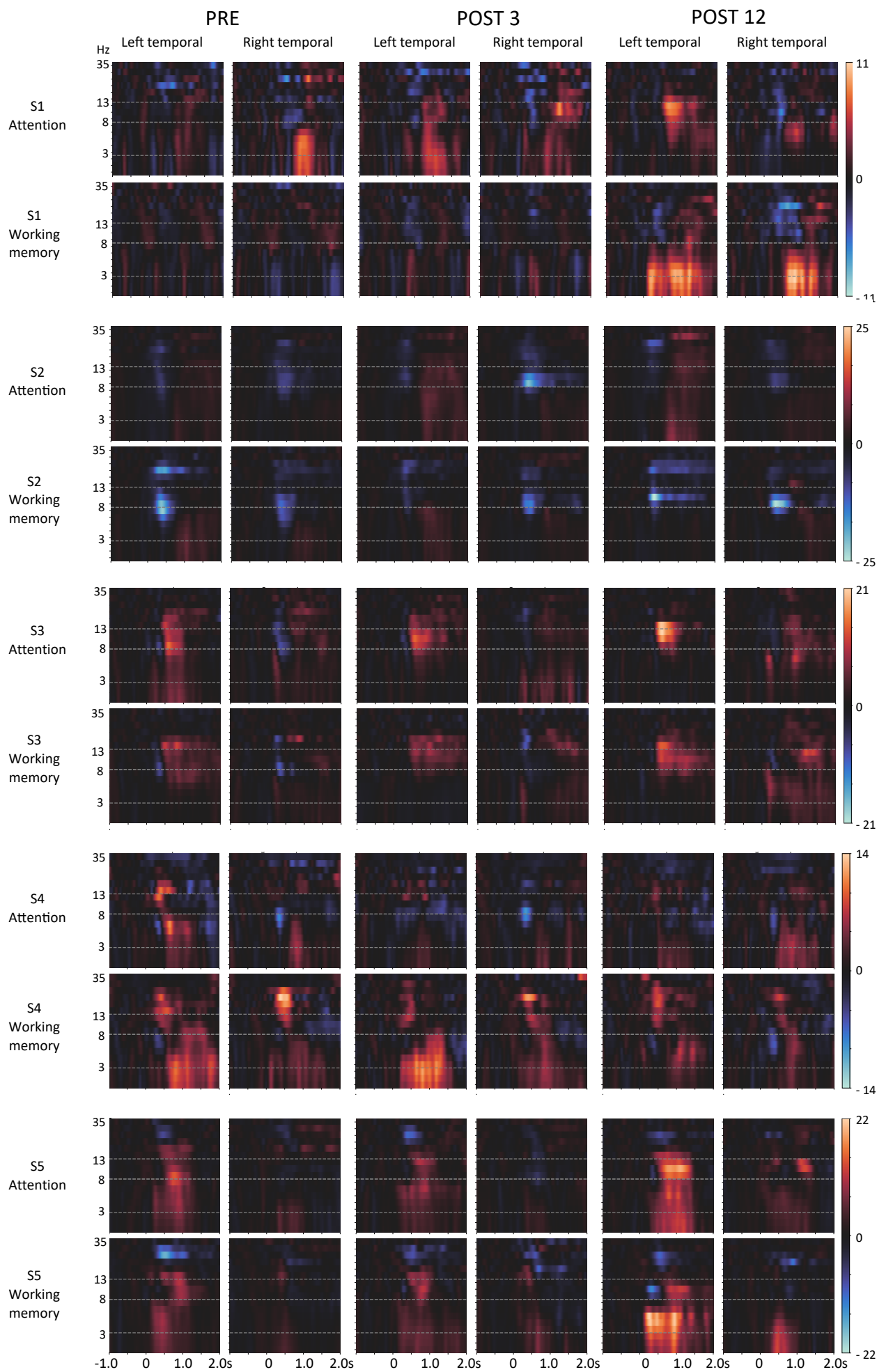

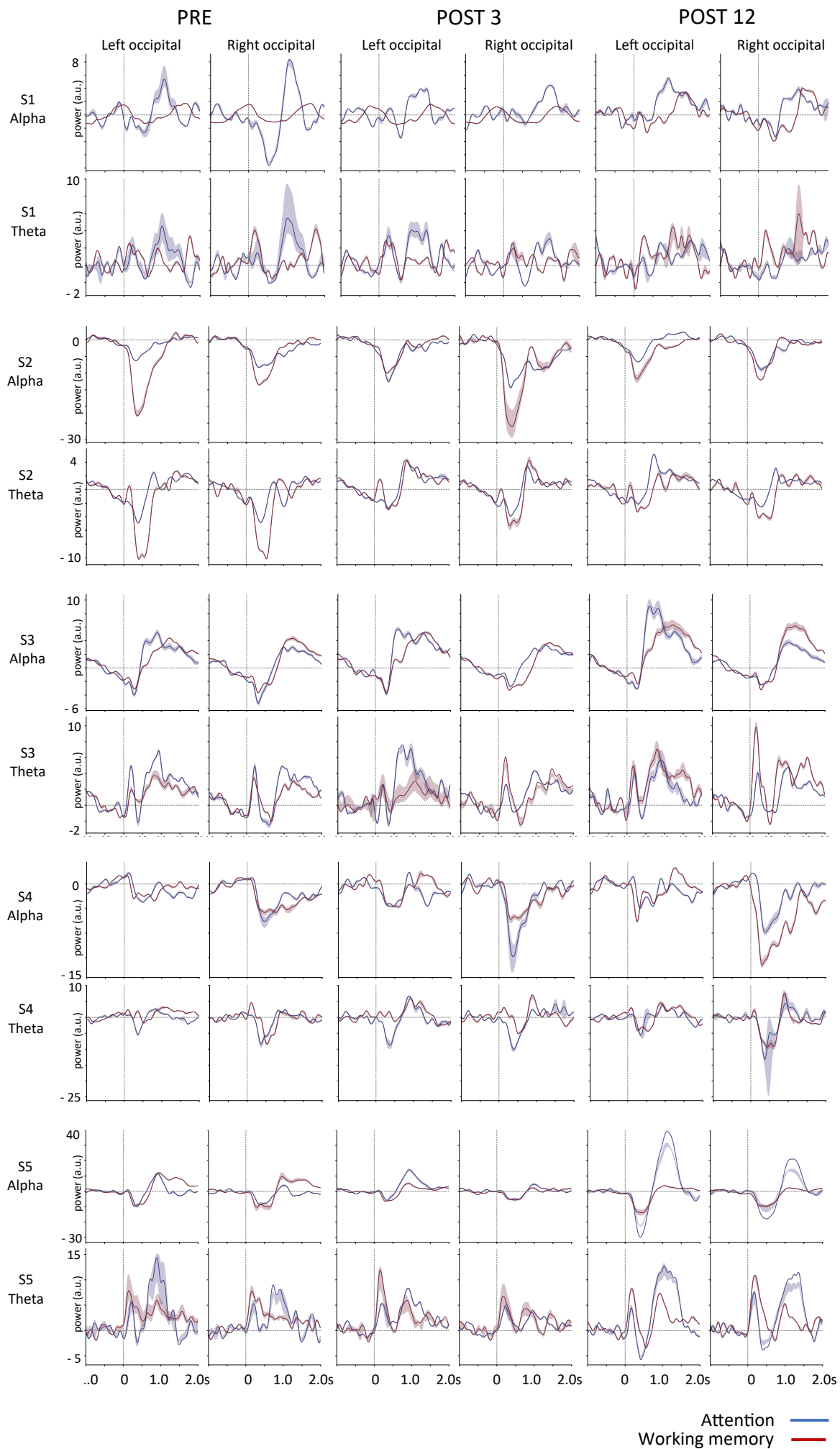

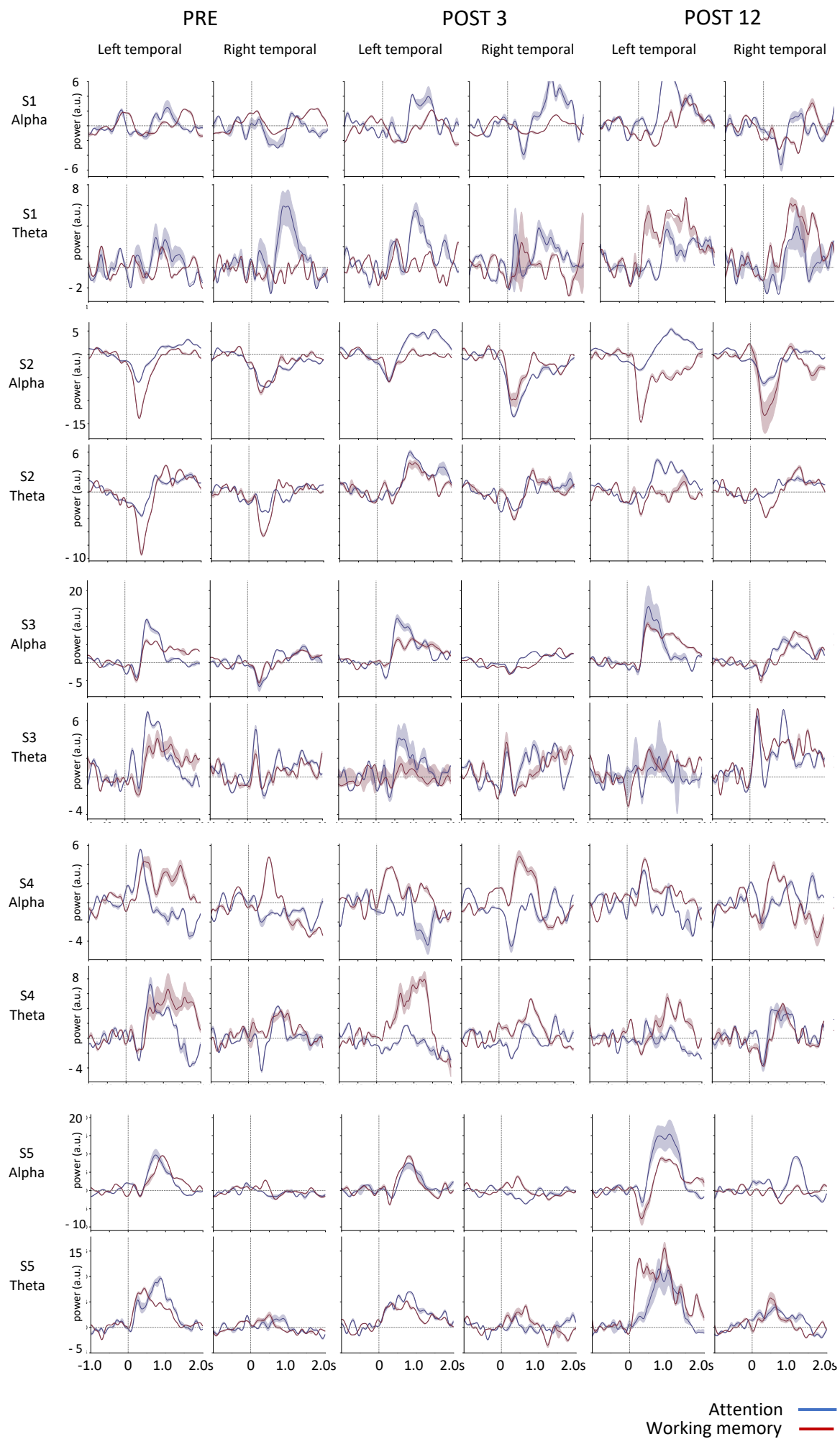

Supplement: S1 Fig — Page 1: TFR maps for individual subjects at PRE, POST3 and POST12 sessions in occipital areas for attention and working memory condition. Page 2: TFR maps for individual subjects at PRE, POST3 and POST12 sessions in temporal areas for attention and working memory condition. Page 3: TSE time-courses for alpha and theta modulation in individual subjects at PRE, POST3 and POST12 sessions in occipital area, overlaid for attention and working memory conditions. Page 4: TSE time-courses for alpha and theta modulation in individual subjects at PRE, POST3 and POST12 sessions in temporal area, overlaid for attention and working memory conditions. (PDF) [file pone.0343689.s001.pdf]
